# The clinicopathologic relevance and prognostic value of tumor deposits and the applicability of N1c category in rectal cancer with preoperative radiotherapy

**DOI:** 10.18632/oncotarget.12058

**Published:** 2016-09-16

**Authors:** Xiao-li Wei, Miao-zhen Qiu, Yi-xin Zhou, Ming-ming He, Hui-yan Luo, Feng-hua Wang, Dong-sheng Zhang, Yu-hong Li, Rui-hua Xu

**Affiliations:** ^1^ Department of Medical Oncology, Sun Yat-sen University Cancer Center, State Key Laboratory of Oncology in South China, Collaborative Innovation Center for Cancer Medicine, Guangzhou 510060, China; ^2^ Department of Pathology, The Johns Hopkins University School of Medicine, Baltimore, MD 21231, USA

**Keywords:** rectal cancer, tumor deposits, preoperative radiotherapy, prognosis, SEER

## Abstract

The clinicopathologic relevance and prognostic value of tumor deposits in colorectal cancer has been widely demonstrated. However, there are still debates in the prognostic value of tumor deposits and the applicability of N1c category in rectal cancer with preoperative radiotherapy. In this study, rectal cancer with preoperative radiotherapy followed by resection of primary tumors registered in Surveillance, Epidemiology and End Results (SEER) database from 2010-2012 were analyzed. There were 4,813 cases eligible for this study, and tumor deposits were found in 514 (10.7%) cases. The presence of tumor deposits was significantly associated with some aggressive characteristics, including poorer tumor differentiation, more advanced ypT category, ypN category and ypTNM stage, distant metastasis, elevated carcinoembryonic antigen, higher positive rates of circumferential resection margin and perineural invasion (all *P* < = 0.001). Tumor deposit was also an independent negative prognostic factor for cancer-specific survival in rectal cancer with preoperative radiotherapy (adjusted *HR* and 95% *CI*: 2.25 (1.51 – 3.35)). N1c category had significant worse survival compared with N0 category (adjusted *HR* and 95% *CI*: 2.41 (1.24 – 4.69)). In conclusion, tumor deposit was a significant and independent prognostic factor, and the N1c category by the 7^th^ edition of AJCC/TNM staging system was applicable in rectal cancer with preoperative radiotherapy.

## INTRODUCTION

Rectal cancer is one of the most common malignancies of the digestive system. Along with colon cancer, it ranks near the top of cancer incidence and cause specific death worldwide [1–3]. Surgery is the only curable treatment for early stage cases [4]. Although some details remain to be explored, preoperative chemoradiotherapy has become the standard treatment strategy for locally advanced rectal cancer [5–10]. Adjuvant chemotherapy does not significantly reduce recurrence, improve disease-free survival (DFS) or overall survival (OS) according to recent studies [11, 12]. Identification of significant prognostic factors helps to determine subgroups with high risk, who may benefit from subsequent systemic chemotherapy.

The value of tumor deposits (TDs) has been widely explored in colorectal cancer. TDs have been reported to be associated with aggressive tumor features, including vascular invasion [13, 14], perineural invasion [13], depth of tumor invasion and regional lymph nodes metastasis [15–17]. In addition, many studies have confirmed an inverse association of TDs with survival in colorectal cancer.

However, in rectal cancer with preoperative radiotherapy, only three studies investigated the prognostic value of TDs and debates existed. The first study specifically evaluating the prognostic value of TDs was conducted by Song JS *et al*. With a retrospective review of 136 rectal cancers staged at ypT3N0M0 after preoperative chemoradiotherapy, they identified TDs in 16 cases. They found no significant differences in both DFS and OS between TDs-negative and TDs-positive cases [18]. By contrast, Gopal P *et al.* analyzed 110 rectal cancers with preoperative chemoradiotherapy, and found TDs to be associated with a trend of higher local recurrence rate and significantly decreased survival [19]. Afterwards, another retrospective study by Zhang LN *et al*. with 310 locally advanced rectal cancer receiving preoperative chemoradiotherapy demonstrated TDs to be a significant negative prognostic factor for DFS and OS [20]. The conclusions were inconsistent and all the studies were of small sample sizes. Thus, we conducted this analysis with a large-sized sample based on Surveillance, Epidemiology and End Results (SEER) database to evaluate the prognostic value of TDs in rectal cancer with preoperative radiotherapy.

## RESULTS

### The relevance of TDs with clinicopathologic characteristics in rectal cancer with preoperative radiotherapy

In general, there were 14,572 rectal adenocarcinoma identified from SEER database in this study. After further selection of cases by the information of surgery and radiotherapy, we obtained 5,439 cases who received preoperative radiotherapy followed by resection of primary rectal cancer. Among them, the information for TDs (Absent / Present) was available in 4,813 (88.5%) cases, and these cases were finally included in this study. TDs were present in 514 (10.7%) cases. The presence of TDs was not associated with gender (Male / Female), age (≤ 59 / > 59 yrs) nor tumor size (≤ 4 / > 4 cm). TDs was present in tumors with more aggressive features, including poorer differentiation, distant metastasis, higher carcinoembryonic antigen (CEA) level, higher rates of circumferential resection margin (CRM) involvement and perineural invasion (all *P* < 0.001, Table 1). In addition, a sequential elevation of positive rate of TDs was presented along with the progression of ypT category, ypN category and ypTNM stage (all *P* < 0.001, Table 1).

**Table 1:** The association of tumor deposits with clinicopathologic characteristics in rectal cancer with preoperative radiotherapy

Characteristics	Tumor deposits N (%)		*P* value
	Absent	Present	
Gender			0.65
Male	2,696 (89.5)	317 (10.5)	
Female	1,603 (89.1)	197 (10.9)	
Age (yrs, median: 59)			0.39
≤ 59	2,146 (88.9)	267 (11.1)	
> 59	2,153 (89.7)	247 (10.3)	
Grade			< 0.001^a^
Well differentiated	257 (91.8)	23 (8.2)	
Moderately differentiated	3,096 (89.7)	355 (10.3)	
Poorly differentiated or undifferentiated	489 (83.2)	99 (16.8)	
Primary tumor size (cm)			0.58
≤ 4	1,927 (88.9)	240 (11.1)	
> 4	1,618 (88.4)	213 (11.6)	
ypT stage			< 0.001^a^
Tis	19 (100.0)	0 (0.0)	
T1	232 (97.9)	5 (2.1)	
T2	586 (95.1)	30 (4.9)	
T3	3,099 (88.5)	404 (11.5)	
T4	362 (82.8)	75 (17.2)	
ypN stage			< 0.001^a^
N0	2,250 (96.7)	76 (3.3)	
N1	1,650 (84.0)	315 (16.0)	
N2	399 (76.4)	123 (23.6)	
Distant metastasis			< 0.001^a^
No	4,075 (90.1)	448 (9.9)	
Yes	224 (77.2)	66 (22.8)	
ypTNM stage			< 0.001^a^
0	19 (100.0)	0 (0.0)	
I	552 (98.9)	6 (1.1)	
II	1,615 (96.3)	62 (3.7)	
III	1,889 (83.3)	380 (16.7)	
IV	224 (77.2)	66 (22.8)	
CEA			< 0.001^a^
Normal	1,635 (91.5)	152 (8.5)	
Borderline	19 (90.5)	2 (9.5)	
Elevated	1,252 (85.3)	215 (14.7)	
CRM			< 0.001^a^
Negative	1,955 (89.8)	221 (10.2)	
Positive	503 (77.9)	143 (22.1)	
Perineural invasion			< 0.001^a^
Negative	3,533 (91.9)	313 (8.1)	
Positive	310 (66.1)	159 (33.9)	

a: Significant *P* value.

Abbreviation: CEA, carcinoembryonic antigen, CRM, circumferential resection margin.

### The prognostic value of TDs in rectal cancer with preoperative radiotherapy

**Figure 1:**
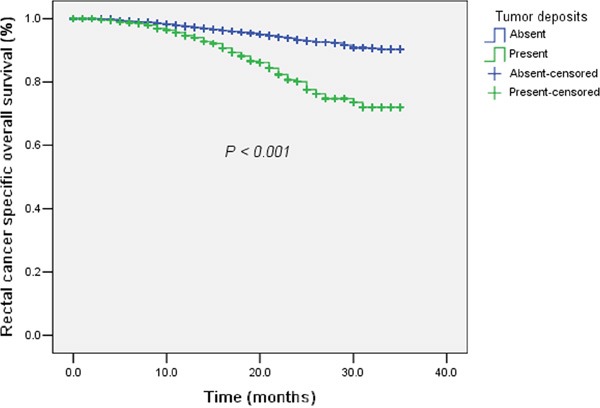
The survival curves of tumor deposits (Absent / Present) plotted by the Kaplan-Meier method in rectal cancer with preoperative radiotherapy Patients with tumor deposits had significantly worse rectal cancer-specific survival compared with those without tumor deposits.

We conducted univariate and multivariate analysis for the 4,813 rectal cancer cases in our study. By univariate analysis, tumor differentiation (Well differentiated / Moderately differentiated / Poorly differentiated or undifferentiated, *P* < 0.001), ypT category (Tis+T1 / T2 / T3 / T4, *P* < 0.001), ypN category (N0 / N1 / N2, *P* < 0.001), distant metastasis (No / Yes, *P* < 0.001), marital status (Widowed / Married / Others, *P* = 0.001), CRM (Negative / Positive, *P* < 0.001), perineural invasion (Negative / Positive, *P* < 0.001) and TDs (Absent / Present, *P* < 0.001) were all found to be significant prognostic factors for rectal cancer-specific survival. All these factors were included for multivariate analysis. Only tumor differentiation (*P* < 0.001), N category (*P* = 0.05), marital status (*P* = 0.03), perineural invasion (*P* = 0.008) and TDs (*P* < 0.001, hazard ratio (*HR*) and 95% confidence interval (*CI*): 2.25 (1.51 – 3.35)) were independent prognostic factors (Table 2). The survival curve of TDs (Absent / Present) plotted by the Kaplan-Meier method were shown in Figure 1.

**Table 2:** Univariate and multivariate analysis for the prognostic value of tumor deposits in rectal cancer with preoperative radiotherapy

characteristics	Univariate analysis				Multivariate analysis		
	No. (%)	*HR*	95% *CI*	*P* value	Adjusted *HR*	95% *CI*	*P* value
Sex		0.87	0.67 – 1.13	0.29			
Male	3013 (62.6)						
Female	1800 (37.4)						
Age (yrs, median: 59)		1.24	0.97 – 1.59	0.08			
≤ 59	2,413 (50.1)						
> 59	2,400 (49.9)						
Grade				< 0.001^a^			< 0.001^a^
Well differentiated	280 (6.5)	1	Reference		1	Reference	
Moderately differentiated	3451 (79.9)	0.81	0.47 – 1.40	0.44	1.07	0.43 – 2.65	0.89
Poorly differentiated or undifferentiated	588 (13.6)	2.93	1.67 – 5.16	< 0.001^a^^b^	3.40	1.34 – 8.60	0.01^a^
ypT stage				< 0.001^a^			0.12
Tis+T1	256 (5.3)	1	Reference		1	Reference	
T2	616 (12.8)	1.45	0.58 – 3.60	0.43	1.26	0.28 – 5.69	0.77
T3	3503 (72.8)	2.39	1.06 – 5.39	0.04	1.31	0.32 – 5.39	0.71
T4	437 (9.1)	5.90	2.54 – 13.74	< 0.001^a^^b^	2.20	0.51 – 9.46	0.29
ypN stage				< 0.001^a^			0.05^a^
N0	2326 (48.2)	1	Reference		1	Reference	
N1	1965 (40.8)	1.63	1.23 – 2.16	0.01^a^^b^	0.95	0.62 – 1.47	0.82
N2	522 (10.8)	3.55	2.55 – 4.93	< 0.001^a^^b^	1.59	0.98 – 2.58	0.06
Distant metastasis		4.47	3.33 – 6.01	< 0.001^a^	3.34	2.17 – 5.13	< 0.001^a^
No	4523 (94.0)						
Yes	290 (6.0)						
Marital status				0.001^a^			0.03^a^
Widowed	354 (7.4)	1	Reference		1	Reference	
Married	2,896 (60.2)	0.53	0.36 – 0.80	0.002^a^^b^	0.57	0.30 – 1.06	0.07
Others	1,563 (32.4)	0.79	0.53 – 1.20	0.27	0.88	0.47 – 1.66	0.70
CRM		2.05	1.47 – 2.85	< 0.001^a^	1.38	0.97 – 1.98	0.09
Negative	2,176 (77.1)						
Positive	646 (22.9)						
Perineural Invasion		3.15	2.34 – 4.24	< 0.001^a^	1.76	1.16 – 2.66	0.008^a^
Negative	3,846 (89.1)						
Positive	469 (10.9)						
Tumor deposits		3.05	2.31 – 4.03	< 0.001^a^	2.25	1.51 – 3.35	< 0.001^a^
Absent	4,299 (89.3)						
Present	514 (10.7)						

a Significant *P* value.

b Significant *P* value after Bonferroni correction.

Abbreviation: CRM, circumferential resection margin. *HR*, hazard ratio, *CI*, confidence interval.

### The prognostic value of TDs in regional lymph nodes negative rectal cancer with preoperative radiotherapy

**Figure 2:**
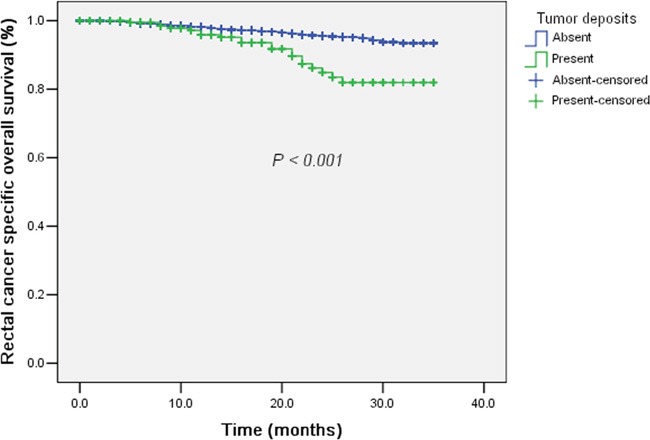
The survival curves of tumor deposits (Absent / Present) plotted by the Kaplan-Meier method in regional lymph nodes negative rectal cancer with preoperative radiotherapy Patients with tumor deposits (categorized as N1c category) had significantly worse rectal cancer-specific survival compared with those without tumor deposits (N0 category).

In the 7^th^ edition of AJCC/TNM staging system, TDs were adopted in regional lymph nodes negative colorectal cancer to establish a subclassification of N1c category. Since we had demonstrated the prognostic value of TDs in rectal cancer with preoperative radiotherapy, it was interesting to analyze the value of N1c category in rectal cancer with preoperative radiotherapy. There were 3,133 cases classified as regional lymph nodes negative among the 4,813 rectal cancer cases. We conducted univariate and multivariate survival analysis to evaluate the prognostic value of TDs in regional lymph nodes negative rectal cancer with preoperative radiotherapy. The results were shown in Table 3. Univariate analysis identified age (≤ 60 / > 60 yrs, *P* = 0.01), tumor differentiation (Well differentiated / Moderately differentiated / Poorly differentiated or undifferentiated, *P* = 0.04), ypT category (Tis+T1 / T2 / T3 / T4, *P* < 0.001), distant metastasis (No / Yes, *P* < 0.001), marital status (Widowed / Married / Others, *P* = 0.01), CRM (Negative / Positive, *P* = 0.01), perineural invasion (Negative / Positive, *P* < 0.001) and TDs (Negative / Positive, *P* < 0.001, Figure 2) to be significant prognostic factor for rectal cancer-specific survival. In multivariate analysis, TDs (*P* = 0.01, *HR* and 95% *CI*: 2.41 (1.24 – 4.69)) remained as an independent prognostic factor. In addition, CRM (*P* = 0.02) and perineural invasion (*P* = 0.03) were also significant prognostic factors. Older patients (> 60 yrs) were found to have a trend of worse survival than younger (*P* = 0.06). Since regional lymph nodes negative cases with TDs was classified as N1c in the 7^th^ AJCC/TNM staging system, our analysis proved the rationale of N1c category in rectal cancer with preoperative radiotherapy.

**Table 3:** Univariate and multivariate analysis for the prognostic value of tumor deposits in regional lymph nodes negative rectal cancer with preoperative radiotherapy

characteristics	Univariate analysis				Multivariate analysis		
	No. (%)	*HR*	95% *CI*	*P* value	Adjusted *HR*	95% *CI*	*P* value
Sex		0.92	0.59 – 1.43	0.89			
Male	1,962 (62.6)						
Female	1,171 (37.4)						
Age (yrs, median: 60)		1.69	1.09 – 2.62	0.01^a^	1.70	0.98 – 2.98	0.06
≤ 60	1,615 (51.5)						
> 60	1,518 (48.5)						
Grade				0.04^a^			0.15
Well differentiated	198 (7.1)	1	Reference		1	Reference	
Moderately differentiated	2,303 (82.7)	0.73	0.33 – 1.60	0.43	0.77	0.23 – 2.52	0.66
Poorly differentiated or undifferentiated	285 (10.2)	1.45	0.59 – 3.60	0.42	1.51	0.41 – 5.61	0.54
ypT stage				< 0.001^a^			0.21
Tis+T1	183 (5.8)	1	Reference		1	Reference	
T2	433 (13.8)	1.36	0.43 – 4.33	0.61	1.77	0.21 – 14.73	0.60
T3	2,271 (72.5)	1.82	0.66 – 5.02	0.25	1.46	0.20 – 10.76	0.71
T4	246 (7.9)	6.13	2.08 – 18.01	0.001^a^^b^	3.04	0.38 – 24.21	0.29
Distant metastasis		4.41	2.34 – 8.31	< 0.001^a^	2.54	1.00 – 6.47	0.05
No	3,013 (96.2)						
Yes	120 (3.8)						
Marital status				0.01^a^			0.60
Widowed	240 (7.7)	1	Reference		1	Reference	
Married	1,871 (59.7)	0.47	0.25 – 0.88	0.01^a^^b^	1.12	0.38 – 3.31	0.83
Others	1,022 (32.6)	0.63	0.33 – 1.20	0.16	1.47	0.49 – 4.42	0.50
CRM		1.95	1.07 – 3.56	0.01^a^	2.00	1.12 – 3.56	0.02^a^
Negative	1,434 (80.1)						
Positive	357 (19.9)						
Perineural Invasion		2.82	1.48 – 5.35	< 0.001^a^	2.21	1.10 – 4.47	0.03^a^
Negative	2,626 (93.1)						
Positive	194 (6.9)						
Tumor deposits		3.34	1.77 – 6.29	< 0.001^a^	2.41	1.24 – 4.69	0.01^a^
Absent	2,916 (93.1)						
Present	217 (6.9)						

a: Significant *P* value.

b: Significant *P* value after Bonferroni correction.

Abbreviation: CRM, circumferential resection margin. *HR*, hazard ratio, *CI*, confidence interval.

## DISCUSSION

We demonstrated the prognostic value of TDs and the rational of N1c category in rectal cancer with preoperative radiotherapy using the SEER database registered from 2010 – 2012 in this study. To our knowledge, this was so far the largest study to investigate the prognostic value of TDs in rectal cancer with preoperative radiotherapy.

Firstly mentioned in the 5^th^ edition of AJCC/TNM staging system, the definition of TDs had evolved along with the release of subsequent editions. During the evolution of TDs definition, the clinicopathologic relevance and prognostic value of TDs had been widely investigated and confirmed in colorectal cancer [13, 15, 17, 21–24]. However, the applicability of TDs in rectal cancer with preoperative chemoradiotherapy had been doubted due to pathological changes induced by chemoradiotherapy. The feature of tumor regression might present with tumor nodules surrounded by fibroinflammatory stroma, which might cause confusion to distinguish residual microfoci and TDs [25, 26]. Thus the value of TDs in rectal cancer with preoperative chemoradiotherapy needed further assessment.

In rectal cancer receiving preoperative radiotherapy, consistent with the findings by Gopal P et al [19] and Zhang LN et al [20], we demonstrated the relevance of TDs with several aggressive tumor features, including more intensive regional lymph nodes metastasis [19, 20], more perineural invasion [19] and higher CEA level [20]. In addition, we also found TDs to be significantly associated with poorer tumor differentiation, more advanced ypT category and ypTNM stage, distant metastasis, as well as higher positive rates of CRM involvement and perineural invasion. It seemed that TDs was not only indicators of more advanced tumor stage, but also associated with intrinsic tumor aggressiveness. Our study also verified the prognostic value of TDs for rectal cancer-specific survival in rectal cancer with preoperative radiotherapy, which was in accordance with the studies by Gopal P et al and Zhang LN et al. Furthermore, by demonstrating the significant and independent prognostic value of TDs in regional lymph nodes negative group, we also proved the applicability of N1c category established by the 7^th^ edition of AJCC/TNM staging system in rectal cancer with preoperative radiotherapy.

Interestingly, in our study, by univariate analysis, although ypT and ypN categories were significant prognostic factors for rectal cancer-specific survival, after adjusting by other prognostic factors, ypT category turned out to be not an independent prognostic factor in rectal cancer with preoperative radiotherapy, and also in cases with negative regional lymph nodes. We further conducted univariate and multivariate analysis for the prognostic value of ypTNM stage (0+I/II/III/IV) in rectal cancer with preoperative radiotherapy (Supplementary Table S1). We found that only stage IV patients had significant different survival compared with stage 0+I patients. This discovery called for a survey of literatures about the applicability of ypTNM stage in rectal cancer with preoperative radiotherapy or preoperative chemoradiotherapy. As a result, we found that it was actually not profoundly researched. Song JS et al proposed the irrelevance of ypTNM stage with DFS and OS in rectal cancer with preoperative chemoradiotherapy [18]. Although several other studies indicated strong association of ypTNM stage and survival in rectal cancer with preoperative chemoradiotherapy, their conclusions were based on univariate analysis [20, 27] or incomplete multivariate analysis [28, 29], for example, adjusted only by age and sex [28]. We didn't find any study to include some important postoperative pathological factors in survival analysis, such as TDs and CRM. Since our study identified these postoperative pathological features to have even more important prognostic value compared with ypT category and ypTNM stage, more comprehensive investigations about the applicability of ypTNM stage in rectal cancer with preoperative radiotherapy were further needed, especially for those without distant metastasis.

Several limitations of our study were noteworthy. Because information of chemotherapy was not available in the SEER database, we could only include rectal cancer with preoperative radiotherapy for analysis in this study. Chemoradiotherapy was the standard treatment for rectal cancer, thus this was one of the major limitations in our study. In addition, several important pathologic factors, including tumor regression grade and vascular invasion were not accessible, thus the conclusions of our study were not adjusted by these important prognostic factors. More importantly, the inter-observer variability in diagnosing TDs was particularly challenging for SEER data due to the various pathologists involved in the data generation. In addition, radiotherapy could increase tissue fibrosis and might cause false-positive diagnoses of TDs. The uniformity of diagnosis and possible false-positive diagnosis were major limitations in our study. These limitations couldn't be resolved and should be particularly noticed in our study with SEER data. Given these limitations, a further verification of our conclusions using a population with more complete information was warranted. In conclusion, tumor deposit was a significant and independent prognostic factor, and N1c category by the 7^th^ edition of AJCC/TNM staging system was applicable in rectal cancer with preoperative radiotherapy.

## MATERIALS AND METHODS

### Ethics statement

This study was deemed exempt from institutional review board approval by Sun Yat-sen University Cancer Center and informed consent was waived. This study was conducted in accordance with the ethical standards of the World Medical Association Declaration of Helsinki.

### SEER database and case selection

The dataset used for analysis in this study was based on the November 2014 data submission “Incidence-SEER 18 Regs Research Data + Hurricane Katrina Impacted Louisiana Cases, Nov 2014 Sub (1973-2012 varying)”. According to the International Classification of Diseases for Oncology, third edition (ICD-O-3) topography codes and histology codes, adenocarcinoma (Code 8140 – 8147, 8210 – 8211, 8220 – 8221, 8255, 8260 – 8263, 8480 – 8481, 8490 and 8574) of rectum (Code C20.9) was included in this study. In addition, we restricted eligibility to patients with records of the 7^th^ American Joint Committee on Cancer/tumor node metastasis (AJCC/TNM) category from 2010 to 2012. We also excluded cases without follow-up records (survival time code of 0 months) and patients with primary tumors other than rectal cancer. Further limitations about radiation (Radiation sequence with surgery) and surgery (RX Summ--Surg Prim Site (1998+)) were also considered for the final study population.

### Information for tumor deposits

Tumor deposits were defined as follows in the 7^th^ edition of AJCC/TNM staging system: “The deposit should be in the pericolorectal fat or adjacent mesocolic fat, it should be away from the leading edge of the tumor, there should be no evidence of residual lymph node tissue, and finally the tumor deposit should be within the lymph drainage area of the primary carcinoma”. This was different from previous definitions which laid more importance on the size and shape of tumor nodules in the 5^th^ and 6^th^ edition of AJCC/TNM staging system. With the implement of Collaborative Stage Data Collection System Version 2 (CSv2) in 2010, the information of TDs was recorded in SEER database as site-specific factor (SSF) 4. The codes and description of the SSFs are available at https://cancerstaging.org/cstage/schema/Pages/version0205.aspx.

### Statistical analysis

We performed all the analyses with SPSS for windows V.13.0. (SPSS Inc., Chicago, IL, USA). The association of TDs with clinicopathologic features was conducted using chi-square test or Kruskal-Wallis H test. Rectal cancer-specific survival was calculated as the time interval between the diagnosis of rectal cancer and the death attributed to rectal cancer, or censored at the death from other causes or the last visit. Univariate and multivariate survival analyses were computed for the prognostic value of TDs for rectal cancer-specific survival. Survival curves were plotted by the Kaplan-Meier method and compared using the log-rank test. *HR* and 95% *CI* were computed with the cox proportional hazards model. A two tailed *P* value < 0.05 was considered statistically significant. Bonferroni correction was applied in univariate analysis.

## SUPPLEMENTARY TABLE


