# Supplementary material for: The clinicopathologic relevance and prognostic value of tumor deposits and the applicability of N1c category in rectal cancer with preoperative radiotherapy
Source: Oncotarget. 2016 Sep 16;7(46):75094–103. doi: 10.18632/oncotarget.12058 (PMC5342725; doi:10.18632/oncotarget.12058)
Supplement: Supplementary file 1 [file oncotarget-07-75094-s001.pdf]

The clinicopathologic relevance and prognostic value of tumor deposits and the applicability of N1c category in rectal cancer with preoperative radiotherapy

SUPPLEMENTARY TABLE

Supplementary Table S1: Univariate and multivariate analysis of the prognostic value of ypTNM stage in rectal cancer with preoperative radiotherapy

Characteristics	Univariate analysis		Multivariate analysis		
	No. (%)	*P* value	Adjusted *HR*	95% *CI*	*P* value
Sex		0.29			
Male	3013 (62.6)				
Female	1800 (37.4)				
Age (yrs, median: 59)		0.08			
≤ 59	2,413 (50.1)				
> 59	2,400 (49.9)				
Grade		< 0.001[a]			< 0.001[a]
Well differentiated	280 (6.5)		1	Reference	
Moderately differentiated	3451 (79.9)		1.07	0.43 – 2.66	0.88
Poorly differentiated or undifferentiated	588 (13.6)		3.75	1.48 – 9.48	0.01[a]
Marital status		0.001[a]			0.02[a]
Widowed	354 (7.4)		1	Reference	
Married	2,896 (60.2)		0.56	0.30 – 1.04	0.07
Others	1,563 (32.4)		0.89	0.48 – 1.66	0.72
CRM		< 0.001[a]	1.42	0.99 – 2.05	0.06
Negative	2,176 (77.1)				
Positive	646 (22.9)				
Perineural Invasion		< 0.001[a]	1.77	1.17 – 2.68	0.01[a]
Negative	3,846 (89.1)				
Positive	469 (10.9)				
Tumor deposits		< 0.001[a]	2.34	1.57 – 3.48	< 0.001[a]
Absent	4,299 (89.3)				
Present	514 (10.7)				
ypTNM stage		< 0.001[a]			< 0.001[a]
0+I	577 (12.0)		1	Reference	
II	1,677 (34.8)		1.35	0.56 – 3.24	0.51
III	2,269 (47.1)		1.38	0.59 – 3.26	0.46
IV	290 (6.0)		4.99	2.03 – 12.26	< 0.001[a]

[a] Significant *P* value.
Abbreviation: CRM, circumferential resection margin. *HR*, hazard ratio, *CI*, confidence interval.
